# An Independent Locus Upstream of *ASIP* Controls Variation in the Shade of the Bay Coat Colour in Horses

**DOI:** 10.3390/genes11060606

**Published:** 2020-05-30

**Authors:** Laura J. Corbin, Jessica Pope, Jacqueline Sanson, Douglas F. Antczak, Donald Miller, Raheleh Sadeghi, Samantha A. Brooks

**Affiliations:** 1 Population Health Sciences, Bristol Medical School, University of Bristol, Bristol BS8 2BN, UK; laura.corbin@bristol.ac.uk; 2 MRC Integrative Epidemiology Unit at University of Bristol, Bristol BS8 2BN, UK; 3 Bristol Veterinary School, University of Bristol, Bristol BS8 1QU, UK; jp14712@bristol.ac.uk; 4 Department of Animal Sciences, University of Florida, Gainesville, FL 32610, USA; jsanson@mail.usf.edu; 5 Baker Institute for Animal Health, College of Veterinary Medicine, Cornell University, Ithaca, NY 14853, USA; dfa1@cornell.edu (D.F.A.); dm96@cornell.edu (D.M.); Rsadeghi2@alumni.ut.ac.ir (R.S.); 6 UF Genetics Institute, University of Florida, Gainesville, FL 32611, USA

**Keywords:** horse, equine, bay, coat colour, ASIP, genome-wide association study

## Abstract

Novel coat colour phenotypes often emerge during domestication, and there is strong evidence of genetic selection for the two main genes that control base coat colour in horses—*ASIP* and *MC1R*. These genes direct the type of pigment produced, red pheomelanin (*MC1R*) or black eumelanin (*ASIP*), as well as the relative concentration and the temporal–spatial distribution of melanin pigment deposits in the skin and hair coat. Here, we describe a genome-wide association study (GWAS) to identify novel genic regions involved in the determination of the shade of bay. In total, 126 horses from five different breeds were ranked according to the extent of the distribution of eumelanin: spanning variation in phenotype from black colour restricted only to the extremities to the presence of some black pigment across nearly all the body surface. We identified a single region associated with the shade of bay ranking spanning approximately 0.5 MB on ECA22, just upstream of the *ASIP* gene (*p =* 9.76 × 10^−15^). This candidate region encompasses the distal 5′ end of the *ASIP* transcript (as predicted from other species) as well as the *RALY* gene. Both loci are viable candidates based on the presence of similar alleles in other species. These results contribute to the growing understanding of coat colour genetics in the horse and to the mapping of genetic determinants of pigmentation on a molecular level. Given pleiotropic phenotypes in behaviour and obesity for *ASIP* alleles, especially those in the 5′ regulatory region, improved understanding of this new *Shade* allele may have implications for health management in the horse.

## 1. Introduction 

Coat colour fulfils three key functions for animals: concealment, communication and the regulation of physiological processes [1]. It follows that many wild animal species are uniformly coloured, with these colours conferring a competitive advantage in camouflage, reproductive success, environmental adaptation and disease tolerance [2]. Yet, while certain coat colours may prove integral to these functions in wild horses, human preferences and demand may result in selection criteria within captive horse populations that favour rare alleles, or may lead to previously unknown phenotypes as a result of selective breeding [3]. By analysing the prevalence of coat colour genes, Ludwig et al. [4] found that variation in coat colour increased rapidly during equine domestication (from 4000 to 3000 BC). Further genetic studies have suggested that this increase in coat colour variation resulted from direct human selection, rather than being due to a relaxation of natural selection pressure [5].

Pigment observed in the skin and hair is produced by the melanocyte in two types: pheomelanin (light/red pigment) versus eumelanin (dark/black pigment) [6,7,8]. Other genetic loci act to influence the differentiation, proliferation and migration of melanocytes resulting in areas lacking pigment, observed as varying degrees of white markings [2]. Genetically, the base coat colour of the horse is controlled by two principal loci: the Extension *(E)* locus at the *melanocortin 1 receptor* (*MC1R*) gene and the Agouti (*A*) locus at the *agouti signalling protein (ASIP)* gene [8,9]. Three known alleles at the *E* locus direct production of black pigment. The dominant or wildtype allele, MC1R*E, permits production of black pigment via the signalling accomplished by the MC1R protein [10]. The most common variant, denoted MC1R*e, corresponds to a missense mutation (S83F) [11]. The resulting loss of function limits black pigmentation, revealing only the underlying red pigment and producing the chestnut colour in a recessive pattern of inheritance. A second, rare recessive allele termed *ea* acts similarly [12]. At the *A* locus, the wildtype allele directs the spatial distribution of black pigment production, restricting this pigment to the extremities of the horse, including the mane and tail. An 11 base pair deletion in the second exon of the *ASIP* coding sequence initiates a frameshift in the ASIP protein, resulting in the recessive black colour (ASIP*a) [10]. *MC1R* alleles interact epistatically with those at *ASIP:* presence of at least one copy of the dominant E^E^ allele at *MC1R*, and the resulting production of black pigment, is required to observe the action of alleles at *ASIP.* Much of what is understood about equine coat colour genetics focuses on this Mendelian inheritance pattern of alleles at the *ASIP* and *MC1R* loci, yet this system does not fully explain the wide range of coat colour shades observed within bay colour horses. 

Given the diversity of phenotypes observed across bay colour horses, we hypothesize that a novel locus modifies the phenotype resulting from known alleles at the *ASIP* and *MC1R* genes. The aim of this study was to conduct a genome-wide association study (GWAS) in order to identify these modifying factors or additional alleles, and to begin to understand the means by which they exert their effects on pigmentation phenotypes.

## 2. Materials and Methods

### 2.1. Sample Cohort

**Figure 1:**
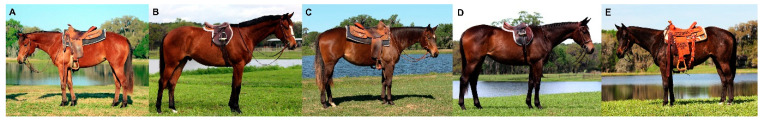
American Quarter Horses illustrating the diversity in shade of bay coat colour, quantified as a ranking within the 129 sampled individuals. The bay shade rank number for each horse is (**A**) 3, (**B**) 27, (**C**) 64, and (**D**) 101 and (**E**) 120.

Photographic images were available for 129 bay horses, representing four different breeds (39 Quarter Horses (QH), 64 Arabians (AR), 23 Persian Horses (PH), 2 Standardbreds (SB), plus one Thoroughbred (TB)). These horses were selected from samples collected for other studies based on an owner-reported “bay” coat colour. We later confirmed that these horses possessed the ASIP*A/a or *A/A* genotype and lacked the MC1R*e/e (chestnut) genotype that could obscure the action of these *ASIP* alleles using linked markers present on the Axiom genotyping array (see “Association analysis” below). Full-body photos for each horse were voluntarily submitted by the horse owner at the time of sample submission for identification purposes, and not specifically for coat colour phenotyping research. As such, photo properties (e.g., season, time of day) and quality were variable. Based on these images, horses were comparatively assigned a ranking within the sampling of 129 images based on the extent of black pigmented areas within the red body coat (Figure 1). Three experienced observers independently ranked the 129 horses. The rankings for each photo were averaged and then the ranking was reassigned based on the linear order of the averages. In two cases of a tie, re-examination of the photos suggested that the shades of colour in these horses were indistinguishable and, therefore, the horses were given the same rank. This produced a quantitative phenotype (rank 1 to 129), whereby low rankings correlated to a light shade of bay with black restricted to the mane, tail and distal limbs, whilst the highest ranked horses possessed black pigment across the entire body, with just light areas in the flank and nose suggesting they were not in fact black in colour (ASIP*a homozygous). 

### 2.2. Genotyping

Genotyping for this study was carried out as part of two previous projects [13,14]. Briefly, blood and/or hair samples were collected from the horses under appropriate animal use approvals (Cornell University 1986–0216, 2008–0121 or 2013–0057 and University of Florida 201408459 or 201708411) and genomic DNA extracted using standard methods. Horses were either genotyped using the Axiom Equine Genotyping Array (*N* = 123, MNEc670k, Affymetrix, Inc., Santa Clara, CA, USA) [15] or genotypes were extracted from whole-genome sequence data presented by Cosgrove et al. (*N* = 6, [14]). Further details of the genotyping and quality control (QC) procedures implemented as part of these projects can be found in Supplementary Methods.

### 2.3. Study-Specific Genotyping Quality Control (QC)

Genotyping QC was performed on data for the sample cohort using Plink (v1.90b4.1) [16,17]. SNPs unassigned to positions on the genome (chromosome “UNK”, 23,427 SNPs) and those not producing genotype calls in at least 95% of samples (200,732 SNPs) were excluded. No filter was applied on the basis of the Hardy–Weinberg Equilibrium (HWE) exact test *p*-value, although this test was performed [18] and results are reported for SNPs of interest. The QC parameters for retaining samples (individual horses) were sample missingness <0.10 and concordance between genotype information and ascertained sex information. In the case of the latter, genotype sex was determined based on X chromosome homozygosity with F estimates smaller than 0.6 yielding female calls and values larger than 0.8 yielding male calls (thresholds were based on a visual inspection of the distribution of F across all samples). Three samples were excluded during QC, leaving 126 horses for the GWAS (39 Quarter Horses (QH), 61 Arabians (AR), 23 Persian Horses (PH), 2 Standardbreds (SB), plus one Thoroughbred (TB)).

More stringent QC thresholds were applied to derive a subset of high-quality autosomal SNPs for use in relatedness testing and population structure analysis. Taking the subset of autosomal SNPs that passed initial QC (described above), further variants were excluded on the basis of SNP missingness > 0.01, MAF < 0.10 and HWE *p* < 1 × 10^−5^. This filtered SNP set comprising 263,649 SNPs was used to infer genetic relatedness by generating a relatedness matrix for all pairs of individuals remaining after QC (using GCTA (Genome-wide Complex Trait Analysis) methodology [19] implemented in Plink). Identity by descent (IBD) was assessed and 0.4 chosen as an appropriate cut off for exclusion in sensitivity analyses. In order to derive an independent subset of SNPs for use in evaluating population structure [20], linkage disequilibrium (LD)-based pruning was then applied using this subset of high-quality autosomal SNPs as input. The LD pruning was conducted in Plink such that, at each step, pairs of variants in each 10 kb window with squared correlation (*r*^2^) greater than 0.1 were noted and variants greedily pruned from the window until no such pairs remained; the window was then shifted using a step size of 5. After pruning, 3259 SNPs were retained and a principal component analysis (PCA) conducted in order to assess population structure within the sample. 

### 2.4. Association Analysis

A GWAS was conducted on the set of SNPs that passed the study-specific QC using linear regression with all analyses implemented in Plink (v1.90b4.1) [16,17]. Ten principal components (PCs) were included in the model to account for population structure within the sample. In addition, horse genotype at two SNPs was conditioned on such that allelic dosage at each variant was fitted as a covariate in the model. The SNPs conditioned on were selected as tagging SNPs for the aforementioned coat colour loci at *ASIP* and *MC1R* [21,22]. The SNP AX-103951024 was used to tag the *ASIP* deletion such that the ‘A’ allele corresponds to the dominant allele, ASIP*A, and the ‘G’ allele to the alternative allele, ASIP*a. The SNP AX-104805525 was used to tag the *MC1R* mutation such that the ‘C’ allele corresponds to the dominant allele, MC1R*E, and the ‘T’ allele to the alternative allele, MC1R*e. Previous evidence suggests that as well as jointly determining bay coat colour, these loci (in particular *MC1R*) may have a dosage effect on shade of bay [8,23]. Genotypes from these two SNPs also confirmed that no chestnut (MC1R*e homozygous) or black (ASIP*a homozygous) horses had inadvertently been included in the sample. 

Adjustment for multiple testing was carried out using the Bonferroni correction, which adjusts the α (*p)* value threshold from *p* = 0.05 to *p* = 0.05/k, where k is the number of statistical tests conducted (i.e., the number of SNPs in the GWAS). This *p*-value was also used in an LD-based clumping procedure carried out on the results in order to identify approximately independent associated loci and their index variants. During this process, GWAS results were grouped into LD-based clumps formed around central index variants, starting with those with the lowest *p*-value. SNPs less than 250 kb away from an index variant and with an *r*^2^ greater than 0.5 with it were assigned to that index variant’s clump. The primary GWAS was re-run further conditioning on the index variants from the first GWAS. In addition, a sensitivity analysis was performed to evaluate the impact on results of highly related horses in the sample. In this analysis, the GWAS was run as in the primary analysis but with one of each pair with IBD > 0.4 excluded.

Further processing of results, including generation of Manhattan and QQ plots, was conducted in R v3.4.1 [24]. Haploview [25] was used to visualize the LD structure in associated regions. A series of linear models were fitted to explore the relationship between associated variants and shade of bay rank within the major breed groups represented in the sample. Linear regression and LD analyses (in Plink) were used to explore the relationship of associated variants with the SNPs used to tag known mutations in *ASIP* and *MC1R.*

### 2.5. Gene Annotation

Gene annotation was examined using the Ensembl and UCSC (University of California, Santa Cruz) Genome Browser databases [26,27]. Although the most current reference genome assembly for the horse is EquCab3.0 [28], we discovered an artificial duplication spanning approximately Chr22:25,700,000–25,870,000, including the region associated with *Shade*, within this assembly (Figure S1). The presence of any large-scale structural variant in this area could not be confirmed by examining assembled haplotypes created using the 10× Genomics Chromium library preparation methods for the reference genome animal (heterozygous for *Shade* associated SNPs across this region) (data from Kalbfleisch et al. (2018) [28] and shared kindly on request). Therefore, EquCab2.0 [29] was used to visualize gene annotation instead of EquCab3.0, and EquCab2.0 coordinates are reported for all polymorphisms.

For the subset of horses with whole-genome sequence data available [14], we investigated potentially functional polymorphisms within a conservative candidate region (chr22:24,841,938–25,178,735) using the Variant Annotation Integrator tool available in the UCSC Genome Browser (set to report non-synonymous and splice region variants) and all annotated Ensembl genes (release 92) [27,30].

### 2.6. Genotyping of RALY Indel Polymorphism

Primers flanking a target indel (chr22:24,916,671–24,917,088) were designed based on the EquCab2.0 reference assembly using Primer3 [31]: RALYx8R-GCGACAGAAGCTGTGTCCTC, RALYx8v2F-[6-FAM]TCCCAGTAAAGAGCAGTGAGC. Genomic DNA was available from just 107 individuals for genotyping, as some individuals did not have sufficient DNA sample remaining. 50 ng genomic DNA, OneTaq® Hot Start ^2×^ Master Mix with Standard Buffer (New England BioLabs Inc., Ipswich, MA, USA) and 0.4 μM of each primer were used for Polymerase Chain Reaction (PCR) with 60 °C in the annealing step (in a 20 µL total volume) according to the manufacturer’s directions. Amplified products were then examined for product size using capillary electrophoresis on an ABI3700 at the Cornell BioResource Center and the resulting electropherograms examined using STRand v2.4 [32]. The multiple indel alleles were coded by calculating the number of inserted base pairs (i.e., 0/0 genotype = 0, 0/12 genotype = 12 and 6/6 genotype = 12). We assessed for an association with shade of bay rank using a linear model including the *ASIP*- and *MC1R*-linked SNP genotypes as covariates, as was done for the GWAS, using JMP 14.1 (SAS Inc., Cary, NC, USA).

## 3. Results

Following QC, the GWAS was performed on 331,084 SNPs in 126 horses. The median (range) of genetic relatedness within the sample was −0.01 (−0.15, 0.67), with 96 and 13 pairs having a genetic relatedness value greater than 0.25 and 0.40, respectively (Supplementary Figure S2). Using an LD-pruned subset of the high-quality autosomal variants used in the genetic relatedness analysis (3259 SNPs), a PCA was conducted. This analysis revealed structure related to the different breed groups present in the sample (Supplementary Figure S3). In an analysis of the *ASIP* (AX-103951024) and *MC1R* (AX-104805525) SNPs with respect to the shade of bay phenotype, a weak positive association was observed between the G allele at AX-103951024 and rank (β = 16.5, se = 8.7, *p* = 0.06, ‘G’ allele frequency = 0.09), whilst a stronger negative association was observed between the T allele at AX-104805525 and rank (β = −23.9, se = 7.1, *p* = 0.001, ‘T’ allele frequency = 0.36). Both these SNPs were subsequently fitted in the linear regression model used for the GWAS.

**Figure 2:**
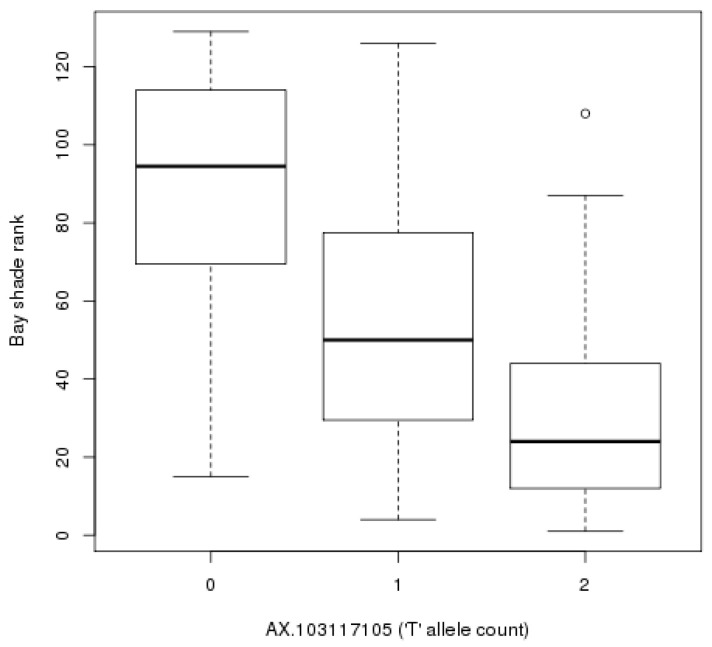
Effect of AX-103117105 on shade of bay. The median bay shade rank for horses in the CC, CT and TT genotype groups was 94.5, 50 and 24, respectively. Upper and lower hinges of boxplots correspond to the first and third quartiles, with the centre line indicating the median and whiskers extending from the hinge to the largest (smallest) value no further than 1.5 x IQR from the hinge; outliers beyond this limit are plotted as unfilled points.

Based on a Bonferroni-adjusted *p*-value of 1.51 × 10^−7^, seven SNPs were associated with the shade of bay phenotype, all within a single locus on chromosome 22 (Supplementary Table S1 and Figure S4). A median-based lambda value of 1.07 suggests minimal inflation of test statistics (Supplementary Figure S5). Following an LD-based clumping procedure, AX-103117105 at 24,998,294 bp on chromosome 22 was the most strongly associated index variant (β = −32.2, 95% confidence interval (CI): −39.4, −25.2, *p* = 9.76 × 10^−15^, effect allele = T, effect allele frequency = 0.38). This effect corresponds to a 32 unit decrease in coat colour shade ranking per additional T allele, which corresponds to approximately one-quarter of the sample size. The effect of genotype at AX-103117105 (with no covariates fitted) is shown in Figure 2. A second SNP in the region, AX-103538677, was the only other index variant identified (β = 27.0, 95% confidence interval (CI): 17.7, 36.4, *p* = 1.22 × 10^−7^, effect allele = C, effect allele frequency = 0.35).

When the GWAS was re-run additionally conditioning on genotype at the index variants, AX-103117105 and AX-103538677, no further SNPs were found to be associated (*p* < 1.51 × 10^−7^). Results from the sensitivity analysis in which highly related horses were removed was largely consistent with the primary GWAS analysis (Supplementary Figure S6). When fitted in a simple linear regression model (without PCs or any other SNPs), genotype at AX-103117105 was associated with shade of bay rank in all three of the major breed groups represented in the sample, as shown in Table 1 and Supplementary Figure S7.

**Figure 3:**
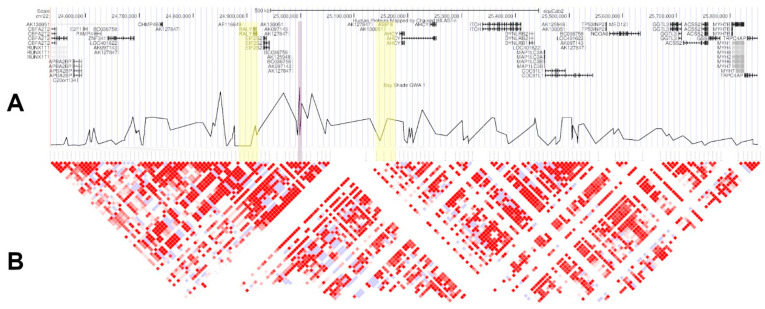
The GWAS-identified candidate region chr22:24,535,302–25,851,386. (**A**) Orthologous human proteins (top) and SNP *p-*values plotted by position (bottom); the best marker from GWAS is highlighted in purple, and the two functional candidate genes *ASIP* and *RALY* in yellow. (**B**) Haploview plot of pairwise LD between SNPs. Relationships where logarithm of odds (LOD) ≥ 2 and D’ = 1 are shaded in red.

The GWAS identified a significant region located on Chromosome 22 and characterized by high average LD (Figure 3), with LD structure appearing consistent across the three major breed groups (Supplementary Figures S8–S10). The lead SNP in this region is unlikely to be causal. Rather, it is likely that it is tagging a form of variation that has had an effect on the coat colour. This candidate region encompasses approximately 1.3 MB and contains 21 annotated genes (Figure 3). Of these, *RALY* (encoding the RALY heterogeneous nuclear ribonucleoprotein) and *ASIP* are the only genes previously implicated in pigment phenotypes, and the lead SNP identified in this study, AX-103117105, is located between these two genes (downstream of *ASIP*, upstream of *RALY)*. There appeared to be no interaction effect on shade of bay rank between genotype at the lead SNP from the GWAS (AX-103117105) and genotype at either of the SNPs tagging known variation at *ASIP* and *MC1R* (see Supplementary Results for further details). Rather, the combined effect of AX-103117105 and AX-104805525 (*MC1R*) on shade rank appeared additive, whilst AX-103951024 (*ASIP*) had a negligible effect on shade when fitted alongside AX-103117105 (see Supplementary Figures S11–S13 and Tables S2 and S3).

**Figure 4:**
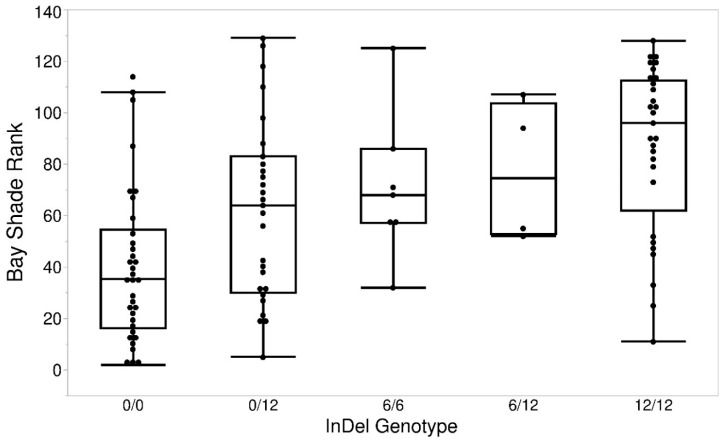
Shade of bay phenotype (rank) differs by genotypes at the *RALY* indel polymorphism. Median ranks for the genotypes, left to right, are as follows: 35.5, 64, 74.5, 68, and 96.

We identified two putative splice region variants, NC_009165.2:g.25167242G > A in *ASIP* and NC_009165.2:g.24916674C > T in *RALY,* but neither is likely to impact splicing as the consensus at these position will accept both nucleotides [32]. Therefore, these were not investigated further as candidate mutations at this *Shade* locus. The known indel responsible for recessive black in the horse was also detected [8]. One polymorphism within *RALY* with a likely impact on protein function was identified: a (GGCAGC)n repeat leading to serine/glycine motif in the amino acid sequence of the sixth exon. Three alleles were clearly present in the animals sequenced by Cosgrove et al. [14]: the reference horse genotype, a deletion of 6 bp (NC_009165.2:g.24,917,006_24,917,007delGGCAGC) and an insertion of 6 bp (NC_009165.2:g.24,917,006_24,917,007insGGCAGC) corresponding to ENSECAP00000010319.1:p.Ser238_Gly239insGS AND delGS in the protein. Although this polymorphism is significantly associated with the shade of bay phenotype (*p* = 3.802 × 10^−10^) (Figure 4), the lead SNP from the GWAS remained the better predictor of the shade phenotype (N = 100 horses genotyped at both loci, AICc (Akaike information criterion with a correction for small sample size) for AX-103117105 = 948.1355, for the *RALY* indel = 967.8614).

## 4. Discussion

GWAS of a ranked bay shade phenotype observed in 126 horses of five diverse breeds identified a single significantly associated region on chromosome 22. The lead variant within the associated region lies between two highly relevant genes for this phenotype—*ASIP* and *RALY.* Working under the assumption that our lead SNP was not causal but tagging a functional mutation within the region, we looked for potentially functional polymorphisms within a conservative candidate region (chr22:24,841,938–25,178,735) from whole-genome sequence data. This revealed a potential candidate causative mutation in *RALY* in the form of a (GGCAGC)_n_ repeat leading to serine/glycine motif in the amino acid sequence of the sixth exon. The *RALY* gene has been linked to the variable black-and-tan colouration seen in certain dog breeds such as Bassett Hounds and Pembroke Welsh Corgis [33]. Whilst the indel did appear to be associated with our shade of bay phenotype, the high LD in the surrounding region meant we were unable to differentiate these effects from those of the SNP identified in the GWAS.

In our data, we observed the expected relationship between shade of bay rank and genotype at the Extension locus in *MC1R* (as reported by [8]), such that horses homozygous for the dominant allele (*E^E^/E^E^*) were on average darker in colour than heterozygotes (*E^E^/E^e^*). The relationship between the Agouti locus and shade of bay rank was much weaker and did not appear to interact with Extension locus variation in this context, as suggested by Druml et al. [23]; it is possible that differences in the frequency of these alleles across breeds could lead to such discrepancies. Conditioning on genotype at both the Agouti and the Extension loci in the GWAS enabled the identification of variants that were independently associated with shade of bay rank. Post hoc analyses were able to provide further evidence that the effect of the lead SNP from the GWAS is independent from any effect of the loci in our sample.

The role of *ASIP* in determining bay coat colour (through its interaction with the Extension locus at *MC1R*) has been known for some time. Given the role of the ASIP protein as an extracellular ligand for the MC1R receptor and an α- melanocyte-stimulating hormone (α-MSH) antagonist [34], it is likely that other mutations within this locus could produce further modifications to coat colour. In dogs for example, the distribution of red or black pigment along the hair shaft or across the body is determined by no less than four known alleles [33]. It is possible, therefore, that intergenic regulatory variants that increase expression of the *ASIP* gene result in a more reddish coat colour, causing the horse to appear a brighter shade of bay. This corresponds to a decrease in shade of bay rank in this study and could suggest the presence of a gain of function variant for the *ASIP* gene in this region. Indeed, diverse structural variants within the distal 5′-untranslated end of the *ASIP* gene are responsible for the *Viable Yellow* series of alleles in mice [35] and the similar phenotype caused by the *Saddle Tan* allele in dogs [33]. In murine *Viable Yellow* alleles, the coat colour and obesity phenotypes of offspring vary dramatically in response to alterations in maternal diet during pregnancy through methylation of *ASIP* regulatory sequences [34]. Thus, epigenetic alterations due to environment could be interesting targets of study for this new equine allele.

The *RALY* gene encodes a heterogeneous nuclear ribonucleoprotein gene, and the protein produced from this gene may affect pre-mRNA splicing and embryonic development [36] with alternate splicing provoking production of multiple transcript variants. Disruption of *RALY* is responsible for the Lethal Yellow phenotype in mice [37] and black-and-tan (on the genetic background of the Saddle Tan haplotype) in dogs [33]. Furthermore, in a recent GWAS of human hair colour involving nearly 300,000 individuals, a SNP in *RALY* (rs6059655) was strongly associated with hair colour, with the same variant apparently acting as an expression quantitative trait locus for *ASIP* in sun-exposed tissues [38]. Combined with the strong LD across this region, functional and regulatory interaction between the *RALY* and *ASIP* loci will make it difficult to disentangle their action in order to discern the precise causative mutations responsible for variation in the shade of bay.

Melanocytes belong to a group of cells that are derived from the neural crest. These “melanoblasts” originate from the neural crest, located on the embryo dorsal surface, before migrating ventrally and maturing to melanocytes [9]. The genes that act on the development and distribution of melanocytes may also affect other ectodermal cells including neurones and glia, as well as potentially affecting mesenchymal cells including bones and cartilages, endocrine cells and vascular smooth muscle cells [39]. This may explain pleiotropic effects between coat colours and disorders in horses, e.g., lethal white foal disease [40], eye problems [41,42] and behaviour [43]. Specifically, the *ASIP* gene has been associated with obesity in mice [44] and somatic malignant melanoma in grey horses [45]. Insulin-mediated glucose transport and adipogenesis are also among the *ASIP*-related pathways [26]. Thus, if this newly described allele alters *ASIP* gene regulation, it may have pleiotropic impacts on health phenotypes like obesity, as is observed in the *Ay* mouse. 

In this work, we made best use of available phenotype, genotype and sequence data to assess the genetic contribution to shade of bay in horses. Whilst there appears to be a relatively strong signal of association in the region of *ASIP* and *RALY*, we were unable to conclusively identify a causal mutation within the candidate region. The difficulty in identifying specific causal variants may in part be due to the structural characteristics of this region of the horse genome. Assembly of this region in EquCab3.0 encountered a heterozygous stretch and misinterpreted this as a large duplication (Supplementary Figure S1). The EquCab2.0 assembly struggled in critical regulatory 5′ and 3′ ends of genes due to high-GC content [28], and may also possess a suspect duplication of *AHCY*, just downstream of *ASIP* (Figure 3). In future efforts, the power of this analysis could be improved with larger sample sizes and by using a more objective measure of coat colour (e.g., use of a spectrophotometer across multiple sites on the body surface, or colour-calibrated photos). In addition, data from a more diverse breed set, together with greater coverage of the genome through partially phased and de novo and individual assemblies, should enable improved resolution in future fine-mapping efforts.

## 5. Conclusions

These results contribute to the growing understanding of coat colour genetics in the horse and to the mapping of genetic determinants of pigmentation on a molecular level. Given pleiotropic phenotypes in behaviour and obesity for *ASIP* alleles, especially those in the regulatory sequence, improved understanding of this new *Shade* allele may have implications for health management in the horse.

## Figures and Tables

**Table 1:** Association of AX-103117105 with shade of bay rank stratified by breed. Results from a linear regression model fitted with shade of bay rank as the dependent variable and genotype at AX-103117105 (T allele count) as the predictor.

Breed Group	Sample Size	β (se)	*p*-Value
All combined	126	−30.0 (3.6)	2.18 × 10^−13^
Arabians	61	−24.1 (5.3)	2.47 × 10^−5^
Persian Horses	23	−33.7 (8.8)	9.95 × 10^−4^
Quarter Horses	39	−41.8 (6.0)	3.00 × 10^−8^

## Supplementary Materials

The following are available online at https://www.mdpi.com/2073-4425/11/6/606/s1, Table S1 List of SNPs on chromosome 22 found to be associated with the shade of bay phenotype in the primary GWAS analysis, Table S2 Haplotype frequencies of AX-103117105 (T/C) and AX-103951024 (G/A) in *ASIP*, Table S3 Linear model results where pairs of SNPs are fitted, Figure S1 Sequence of the candidate region on chromosome 22 showing artificial duplication in EquCab3.0, Figure S2 Histogram of pairwise genetic relatedness estimates, Figure S3 Evidence of population structure in results from a principal component analysis (PCA), Figure S4 Manhattan plot showing primary GWAS result; Figure S5 QQ plot showing primary GWAS result, Figure S6 Results from a sensitivity analysis in which the GWAS was conducted after having excluded one of each pair whose genetic relatedness was estimated to be >0.4, Figure S7 Effect of AX-103117105 on shade of bay by breed group, Figure S8 Haploview plot [5] of associated region using AR samples (N = 61), Figure S9 Haploview plot [5] of associated region using PH samples (N = 23), Figure S10 Haploview plot [5] of associated region using QH samples (N = 39), Figure S11 The combined effect of AX-103117105 (T/C) and AX-103951024 (A/G) genotypes on shade of bay, Figure S12 The combined effect of AX-103117105 (T/C) and AX-104805525 (T/C) genotypes on shade of bay, and Figure S13 The combined effect of AX-104805525 (T/C) and AX-103951024 (A/G) genotypes on shade of bay.

## References

[1] Klungland H., Vage D. (2000). Molecular Genetics of Pigmentation in Domestic Animals. Curr. Genom..

[2] Rieder S. (2009). Molecular tests for coat colours in horses. J. Anim. Breed. Genet..

[3] Seiji M., Shimao K., Birbeck M.S.C., Fitzpatrick T.B. (1963). Subcellular Localisation of Melanin Biosynthesis. Ann. N. Y. Acad. Sci..

[4] Ludwig A., Pruvost M., Reissmann M., Benecke N., Brockmann G.A., Castaños P., Cieslak M., Lippold S., Llorente L., Malaspinas A.S. (2009). Coat color variation at the beginning of Horse domestication. Science.

[5] Fang M., Larson G., Soares Ribeiro H., Li N., Andersson L. (2009). Contrasting Mode of Evolution at a Coat Color Locus in Wild and Domestic Pigs. PLoS Genet..

[6] Tobin D.J. (2008). Human hair pigmentation–Biological aspects. Int. J. Cosmet. Sci..

[7] Prota G. (1980). Recent advances in the chemistry of melanogenesis in mammals. J. Investig. Dermatol..

[8] Rieder S., Taourit S., Mariat D., Langlois B., Guérin G. (2001). Mutations in the agouti (ASIP), the extension (MCIR), and the brown (TYRP1) loci and their association to coat color phenotypes in horses (Equus caballus). Mamm. Genome.

[9] Dreger D. (2012). Gene Interactions with Agouti Signaling Protein Produce Complex Pigmentation Phenotypes in the Domestic Dog. Doctoral Dissertation.

[10] Daverio M.S., Rigalt F., Romero S., Vidal-Rioja L., Di Rocco F. (2016). Polymorphisms in MC1R and ASIP genes and their association with coat color phenotypes in llamas (Lama glama). Small Rumin. Res..

[11] Marklund L., Moller M.J., Sandberg K., Andersson L. (1996). A missense mutation in the gene for melanocyte-stimulating hormone receptor (MC1R) is associated with the chestnut coat color in horses. Mamm. Genome.

[12] Wagner H.J., Reissmann M. (2000). New polymorphism detected in the horse MC1R gene. Anim. Genet..

[13] Patterson Rosa L., Walker N.L., Mallicote M.M., MacKay R.J., Brooks S.A. (2020). Genomics of Congenital Idiopathic Anhidrosis in the Stock-Type Horse. Equine Vet. J..

[14] Cosgrove E.J., Sadeghi R., Schlamp F., Holl H.M., Moradi-Shahrbabak M., Miraei-Ashtiani S.R., Abdalla S., Shykind B., Troedsson M., Stefaniuk-Szmukier M. (2020). Genome diversity and the origin of the Arabian horse. Sci. Rep..

[15] Schaefer R.J., Schubert M., Bailey E., Bannasch D.L., Barrey E., Bar-Gal G.K., Brem G., Brooks S.A., Distl O., Fries R. (2017). Developing a 670k genotyping array to tag ~2M SNPs across 24 horse breeds. BMC Genom..

[16] Chang C., Chow C., Tellier L., Vattikuti S., Purcell S., Lee J. (2015). Second-generation PLINK: Rising to the challenge of larger and richer datasets. Gigascience.

[17] PLINK v1.90.b4.1 2017. https://www.cog-genomics.org/plink/1.9/.

[18] Wigginton J.E., Cutler D.J., Abecasis G.R. (2005). A note on exact tests of Hardy-Weinberg equilibrium. Am. J. Hum. Genet..

[19] Yang J., Lee S.H., Goddard M.E., Visscher P.M. (2011). GCTA: A Tool for Genome-wide Complex Trait Analysis. Am. J. Hum. Genet..

[20] Abdellaoui A., Hottenga J.J., De Knijff P., Nivard M.G., Xiao X., Scheet P., Brooks A., Ehli E.A., Hu Y., Davies G.E. (2013). Population structure, migration, and diversifying selection in the Netherlands. Eur. J. Hum. Genet..

[21] Shang S., Yu Y., Zhao Y., Dang W., Zhang J., Qin X., Irwin D.M., Wang Q., Liu F., Wang Z. (2019). Synergy between MC1R and ASIP for coat color in horses (Equus caballus). J. Anim. Sci..

[22] MacKowski M., Wodas L., Brooks S.A., Cieslak J. (2019). TBX3 and ASIP genotypes reveal discrepancies in officially recorded coat colors of Hucul horses. Animal.

[23] Druml T., Grilz-Seger G., Horna M., Brem G. (2018). Discriminant Analysis of Colour Measurements Reveals Allele Dosage Effect of ASIP/MC1R in Bay Horses. Czech J. Anim. Sci..

[24] (2017). R Core Team R: A Language and Environment for Statistical Computing.

[25] Barrett J.C., Fry B., Maller J., Daly M.J. (2005). Haploview: Analysis and visualization of LD and haplotype maps. Bioinformatics.

[26] Zerbino D.R., Achuthan P., Akanni W., Amode M.R., Barrell D., Bhai J., Billis K., Cummins C., Gall A., Girón C.G. (2018). Ensembl 2018. Nucleic Acids Res..

[27] Kent W.J., Sugnet C.W., Furey T.S., Roskin K.M., Pringle T.H., Zahler A.M., Haussler D. (2002). The Human Genome Browser at UCSC. Genome Res..

[28] Kalbfleisch T.S., Rice E.S., DePriest M.S., Walenz B.P., Hestand M.S., Vermeesch J.R., O′Connell B.L., Fiddes I.T., Vershinina A.O., Saremi N.F. (2018). Improved reference genome for the domestic horse increases assembly contiguity and composition. Commun. Biol..

[29] Wade C.M., Giulotto E., Sigurdsson S., Zoli M., Gnerre S., Imsland F., Lear T.L., Adelson D.L., Bailey E., Bellone R.R. (2009). Genome sequence, comparative analysis, and population genetics of the domestic horse. Science.

[30] Pruitt K.D., Brown G.R., Hiatt S.M., Thibaud-Nissen F., Astashyn A., Ermolaeva O., Farrell C.M., Hart J., Landrum M.J., McGarvey K.M. (2014). RefSeq: An update on mammalian reference sequences. Nucleic Acids Res..

[31] Untergasser A., Cutcutache I., Koressaar T., Ye J., Faircloth B.C., Remm M., Rozen S.G. (2012). Primer3-new capabilities and interfaces. Nucleic Acids Res..

[32] Mucaki E.J., Shirley B.C., Rogan P.K. (2013). Prediction of Mutant mRNA Splice Isoforms by Information Theory-Based Exon Definition. Hum. Mutat..

[33] Dreger D.L., Parker H.G., Ostrander E.A., Schmutz S.M. (2013). Identification of a mutation that is associated with the saddle tan and black-and-tan phenotypes in Basset Hounds and Pembroke Welsh Corgis. J. Hered..

[34] Bultman S.J., Michaud E.J., Woychik R.P. (1992). Molecular characterization of the mouse agouti locus. Cell.

[35] Cropley J.E., Suter C.M., Beckman K.B., Martin D.I.K. (2006). Germ-Line Epigenetic Modification of the Murine A vy allele by Nutritional Supplementation. Proc. Natl. Acad. Sci. USA.

[36] O’Leary N.A., Wright M.W., Brister J.R., Ciufo S., Haddad D., McVeigh R., Rajput B., Robbertse B., Smith-White B., Ako-Adjei D. (2016). Reference sequence (RefSeq) database at NCBI: Current status, taxonomic expansion, and functional annotation. Nucleic Acids Res..

[37] Michaud E.J., Bultman S.J., Klebig M.L., Van Vugt M.J., Stubbs L.J., Russell L.B., Woychik R.P. (1994). A Molecular Model for the Genetic and Phenotypic Characteristics of the Mouse Lethal Yellow (AY) Mutation. Proc. Natl. Acad. Sci. USA.

[38] Hysi P.G., Valdes A.M., Liu F., Furlotte N.A., Evans D.M., Bataille V., Visconti A., Hemani G., McMahon G., Ring S.M. (2018). Genome-wide association meta-analysis of individuals of European ancestry identifies new loci explaining a substantial fraction of hair color variation and heritability. Nat. Genet..

[39] Dupin E., Sommer L. (2012). Neural crest progenitors and stem cells: From early development to adulthood. Dev. Biol..

[40] Lightbody T. (2002). Foal with Overo lethal white syndrome born to a registered quarter horse mare. Can. Vet. J..

[41] Bellone R.R., Holl H., Setaluri V., Devi S., Maddodi N., Archer S., Sandmeyer L., Ludwig A., Foerster D., Pruvost M. (2013). Evidence for a Retroviral Insertion in TRPM1 as the Cause of Congenital Stationary Night Blindness and Leopard Complex Spotting in the Horse. PLoS ONE.

[42] Andersson L.S., Wilbe M., Viluma A., Cothran G., Ekesten B., Ewart S., Lindgren G. (2013). Equine Multiple Congenital Ocular Anomalies and Silver Coat Colour Result from the Pleiotropic Effects of Mutant PMEL. PLoS ONE.

[43] Jacobs L.N., Staiger E.A., Albright J.D., Brooks S.A. (2016). The MC1R and ASIP coat color loci may impact behavior in the horse. J. Hered..

[44] Klebig M.L., Wilkinson J.E., Geisler J.G., Woychik R.P. (1995). Ectopic expression of the agouti gene in transgenic mice causes obesity, features of type II diabetes, and yellow fur. Proc. Natl. Acad. Sci. USA.

[45] Curik I., Druml T., Seltenhammer M., Sundström E., Pielberg G.R., Andersson L., Sölkner J. (2013). Complex Inheritance of Melanoma and Pigmentation of Coat and Skin in Grey Horses. PLoS Genet..

