# Supplementary material for: An Independent Locus Upstream of ASIP Controls Variation in the Shade of the Bay Coat Colour in Horses
Source: Genes (Basel). 2020 May 30;11(6):606. doi: 10.3390/genes11060606 (PMC7349280; doi:10.3390/genes11060606)
Supplement: Supplementary file 1 [file genes-11-00606-s001.pdf]

genes

MDPI

Supplementary Information for

An Independent Locus Upstream of *ASIP* Controls Variation in the Shade of the Bay Coat Colour in Horses

Supplementary Methods

Genotyping

Genotyping for this study was carried out as part of two previous projects [1,2]. Briefly, blood and/or hair samples were collected from the horses in accordance with The Institutional Animal Care and Use Committee (IACUC, Study #201708411) and under appropriate Animal Use approvals as reported in each study. Genomic DNA was extracted using standard methods and all horses genotyped using the Axiom Equine Genotyping Array (MNEc670k, Affymetrix, Inc.) [3] at either GeneSeek® (Neogen Corporation©, Lincoln, NE, USA) or Affymetrix (part of Thermo Fisher Scientific, Santa Clara, CA, USA). Raw CEL files from each genotyping lab were individually loaded into the Axiom Analysis Suite for SNP calling. Sample filters were set to a DishQC score ≥0.82 and a sample call rate ≥90%.

Post genotyping, the variant list was restricted to "PolyHighResolution" clusters with a Fisher's Linear Discriminant of 3.6. Genotypes were then exported to a numeric genotype call format and converted to the Variant Call Format (VCF). Genotype calls from each lab were combined sequentially, with Affymetrix and GeneSeek® calls merged based upon filters set to 90% SNP genotyping rate and 1% minor allele frequency (MAF). A subset of US Arabians (from unselected origins) was used to test all SNPs for Hardy Weinberg Equilibrium (HWE). Autosomal variants with p-values more extreme than 0.005 (corrected for multiple testing) were removed from the genotype files.

Of the 670k SNPs on the array, 114,757 were removed during these pre-processing steps, with the remaining 555,243 comprising the set of SNPs available for our analysis of coat colour. The SNPs were distributed across 31 autosomes, the X chromosome and two groups of unassigned variants.

A subset of the horses underwent whole-genome sequencing [2]. SNPs derived from sequencing of these horses were merged with the genotyped sample dataset to generate the final set of variant calls for downstream analysis. Variants with a genotyping rate < 80%, MAF < 1%, or variants that were flagged as multi-allelic were removed from this final dataset.

Supplementary Results

Combined Effect of Lead SNP and Known Variation in ASIP and MC1R

It has been hypothesised that the Extension (*E*) locus at *MC1R* has a dosage effect on the shade of bay such that horses with the E^E/E^E genotype are darker than those with the E^E/E^e genotype [4]. Whilst in the same study, no evidence was found to support any relationship between the Agouti (*A*) locus and shade of bay, more recent work by Druml et al. [5] suggested that horses carrying the combination of E^E/E^E and A^A/A^a were characterised by darker shades. We conditioned the GWAS on tag SNPs representing the *E* and *A* loci and can, therefore, be fairly confident that the associations we detected are independent of these previously characterised variants. However, in the interests of transparency and completeness, we (1) calculated linkage disequilibrium (LD) and explored the haplotypic background of the lead SNP and the *A* locus (since these are co-located on ECA22); (2) fitted a series of linear regression models to explore further the possibility of overlapping and/or interacting signals.

Linkage disequilibrium results for ASIP

The estimated r^2 between the lead SNP, AX-103117105 (T/C), and the SNP tagging the *A* locus in *ASIP,* AX-103951024 (G/A), was 0.06, and the corresponding D prime estimate was 1. The in-phase alleles were predicted to be TA/CG and the observed and expected allele frequencies are shown in Table S2. Whilst this information is useful in understanding the relationship between these two variants within our dataset, our sample does not constitute a random sample from the population (and has in fact been selected based on genotype at the Agouti locus itself) and, therefore, the haplotypic structure we observe is not likely to be representative of that observed in the population more generally.

Multi-SNP linear model results

In addition to the single-SNP models described in the main text, we fitted three linear models in which the shade of bay rank was the dependent variable and two SNPs (and their interaction term) were fitted as predictors: (1) AX-103117105 (T/C) (lead SNP) and AX-103951024 (G/A) (*A* locus tag SNP); (2) AX-103117105 (T/C) (lead SNP) and AX-104805525 (C/T) (*E* locus tag SNP); (3) AX-103951024 (G/A) (*A* locus tag SNP) and AX-104805525 (C/T) (*E* locus tag SNP). These models revealed no evidence for an interaction between any of the SNP pairs tested (Table S3). The *A* locus, as proxied by AX-103951024, had a negligible effect on the shade of bay when fitted alongside the lead SNP from the GWAS (Table S3, Figure S11). The *E* locus had a dosage effect as previously proposed such that the SNP genotype proxying E^E/E^E was associated with a darker coat colour. The combined effect of the lead SNP from the GWAS and the *E* locus suggested an additive relationship such that horses with the CC genotype at AX-104805525 and the CC genotype at AX-103117105 were (on average) the darkest, whereas those with the CT genotype at AX-104805525 and the TT genotype at AX-103117105 were the lightest (Table S3, Figure S12). There was no evidence to support an interaction between the *E* locus and the *A* locus with respect to shade of bay rank (Table S3, Figure S13). Results from models in which the ten principal components were also fitted as predictors (as in the GWAS) were consistent with the SNP only models (results not shown).

Supplementary Tables

Table S1. List of SNPs on chromosome 22 found to be associated with shade of bay phenotype in the primary GWAS analysis. Results for index SNPs identified using an LD-based clumping procedure are highlighted in bold.

SNP.	BP	EA	Beta (SE)	P-value	EAF	HWE *p*-value
**AX-103538677**	**24687615**	**C**	**27.02 (4.78)**	**1.22×10^{-07}**	**0.35**	**0.84**
AX-104169687	24850601	T	−31.29 (3.71)	1.37×10^{-13}	0.38	0.13
AX-103445116	24968297	G	-31.04 (3.75)	3.09×10^{-13}	0.37	0.18
**AX-103117105**	**24998294**	**T**	**−32.27 (3.61)**	**9.76×10^{-15}**	**0.38**	**0.13**
AX-103727960	25002255	T	31.20 (4.34)	8.10×10^{-11}	0.48	0.60
AX-104760472	25020920	C	−31.96 (3.89)	3.92×10^{-13}	0.35	0.12
AX-103065495	25226879	G	-28.08 (4.34)	$2. \times 10^{-09}$	0.37	0.35

BP = base per position (EquCab2.0); EA = effect allele; SE = standard error; EAF = effect allele frequency; HWE = Hardy Weinberg equilibrium; LD = linkage disequilibrium.

Table S2. Haplotype frequencies of AX-103117105 (T/C) and AX-103951024 (G/A) in *ASIP*. The SNP AX-103117105 (T/C) is the lead SNP from the GWAS. The SNP AX-103951024 (G/A) was used to tag the Agouti locus such that the 'A' allele at AX-103951024 corresponds to the dominant allele, A^A, and the 'G' allele at AX-103951024 to the alternative allele, A^a.

Haplotype	Observed Frequency	Expectation under Linkage Equilibrium
TG	0	0.03
CG*	0.09	0.06
TA*	0.38	0.35
CA	0.53	0.56

* In-phase haplotypes.

Table S3. Linear model results where pairs of SNPs are fitted. The SNP AX-103117105 (T/C) is the lead SNP from the GWAS. The SNP AX-103951024 (G/A) was used to tag the Agouti locus such that the 'A' allele at AX-103951024 corresponds to the dominant allele, A^A, and the 'G' allele at AX-103951024 to the alternative allele, A^a. The SNP AX-104805525 was used to tag the Extension locus such that the 'C' allele at AX-104805525 corresponds to the dominant allele, E^E, and the 'T' allele to the alternative allele, E^e. Linear model results presented as the effect (beta) per additional reference allele with corresponding standard error (se) and p-value.

		SNP1 Effect		SNP2 Effect		Interaction (SNP1*SNP2) Effect	
**SNP 1 (ref. allele*)**	**SNP2 (ref. allele*)**	**Beta (se)**	***p*-value**	**Beta (se)**	***p*-value**	**Beta (se)**	***p*-value**
AX-103117105 (T)	AX-103951024 (G)	−29.8 (3.8)	2.97×10^{-12}	5.4 (10.5)	0.61	5.6 (13.5)	0.68
AX-103117105 (T)	AX-104805525 (T)	−28.8 (5.8)	2.29×10^{-06}	−24.1 (8.0)	3.06×10^{-03}	−2.91 (7.1)	0.68
AX-104805525 (T)	AX-103951024 (G)	−28.4 (7.6)	3.02×10^{-04}	−0.3 (17.0)	0.99	24.0 (19.5)	0.22

* the ref. (reference allele) is the allele being counted to give the 0,1,2 format genotype used in the linear models.

Supplementary Figures

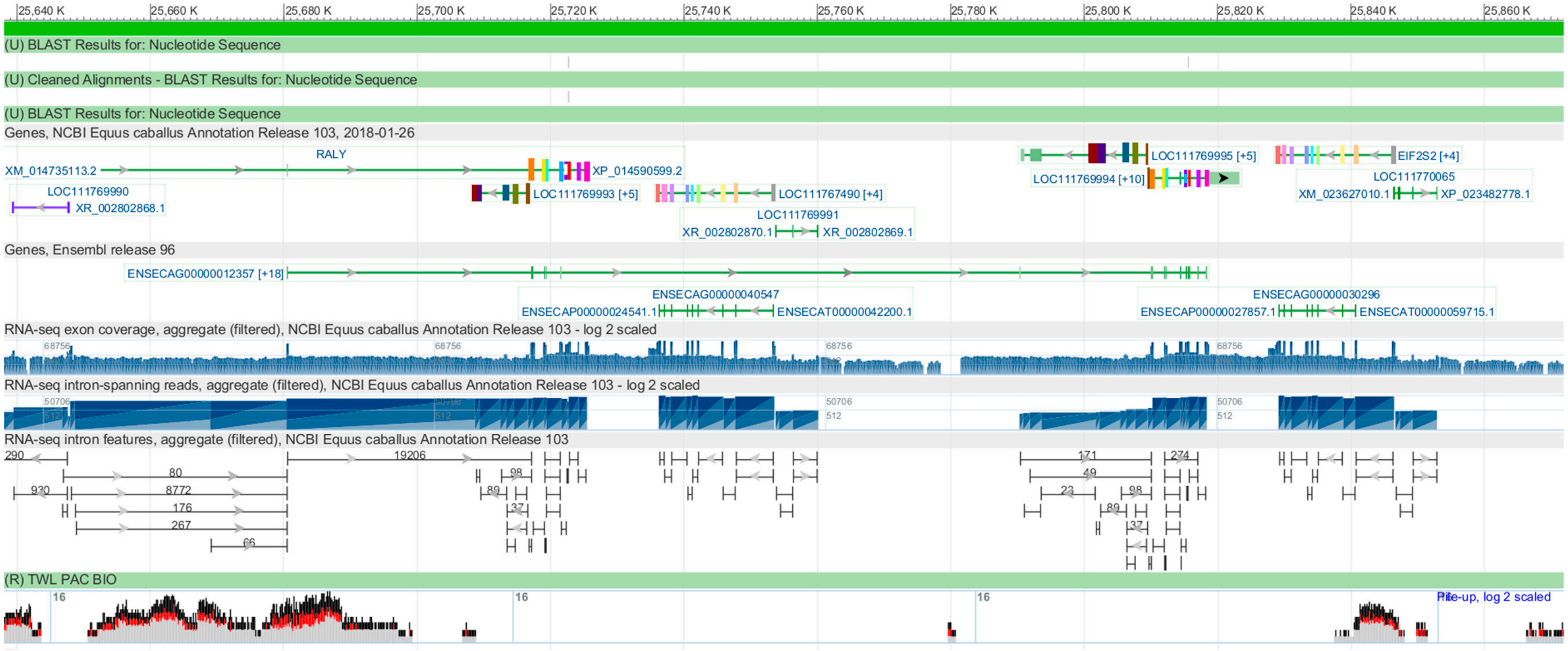

Figure S1. Sequence of *RALY* region on chromosome 22 showing artificial duplication in EquCab3.0.

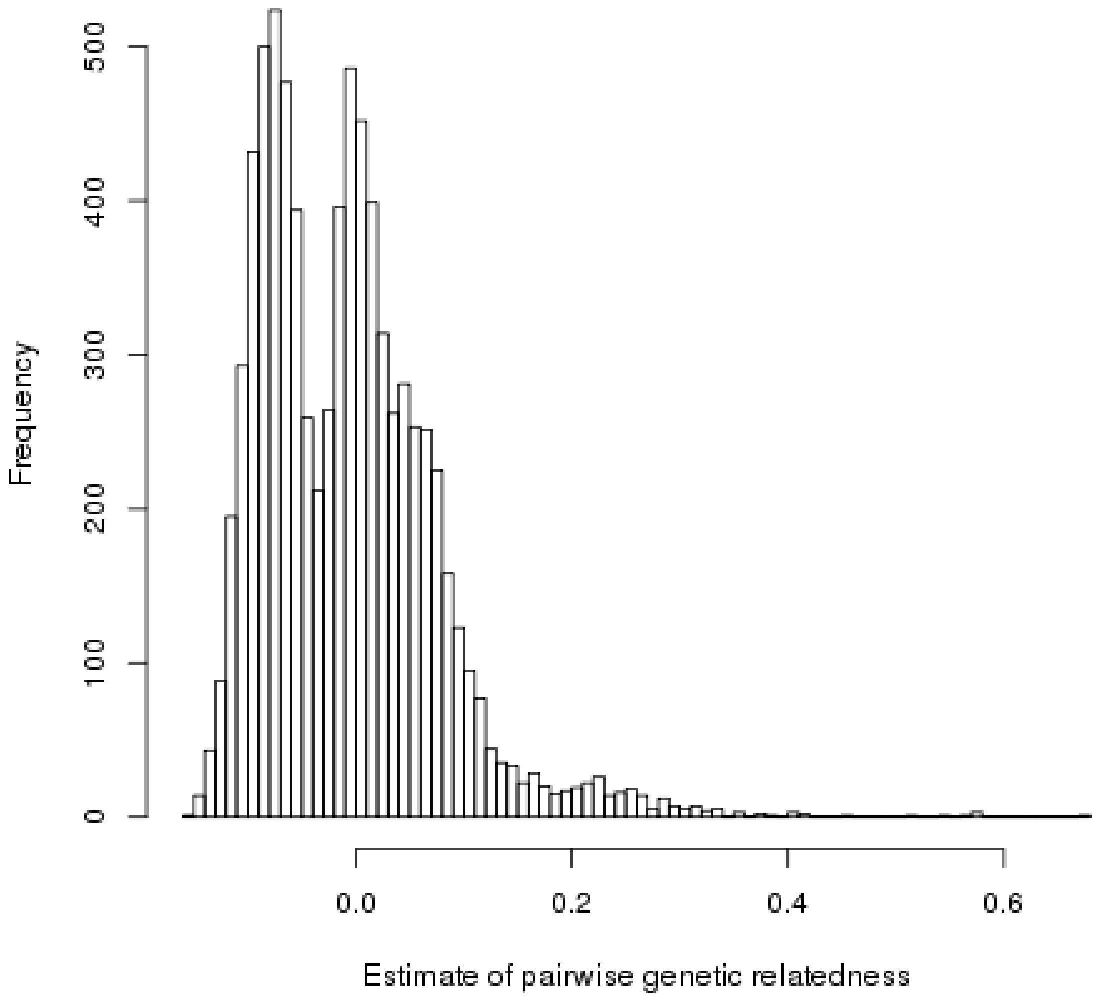

Figure S2. Histogram of pairwise genetic relatedness estimates. Estimates of pairwise identity by descent (IBD) derived using a GCTA (Genome-wide Complex Trait Analysis) approach [6] applied in Plink.

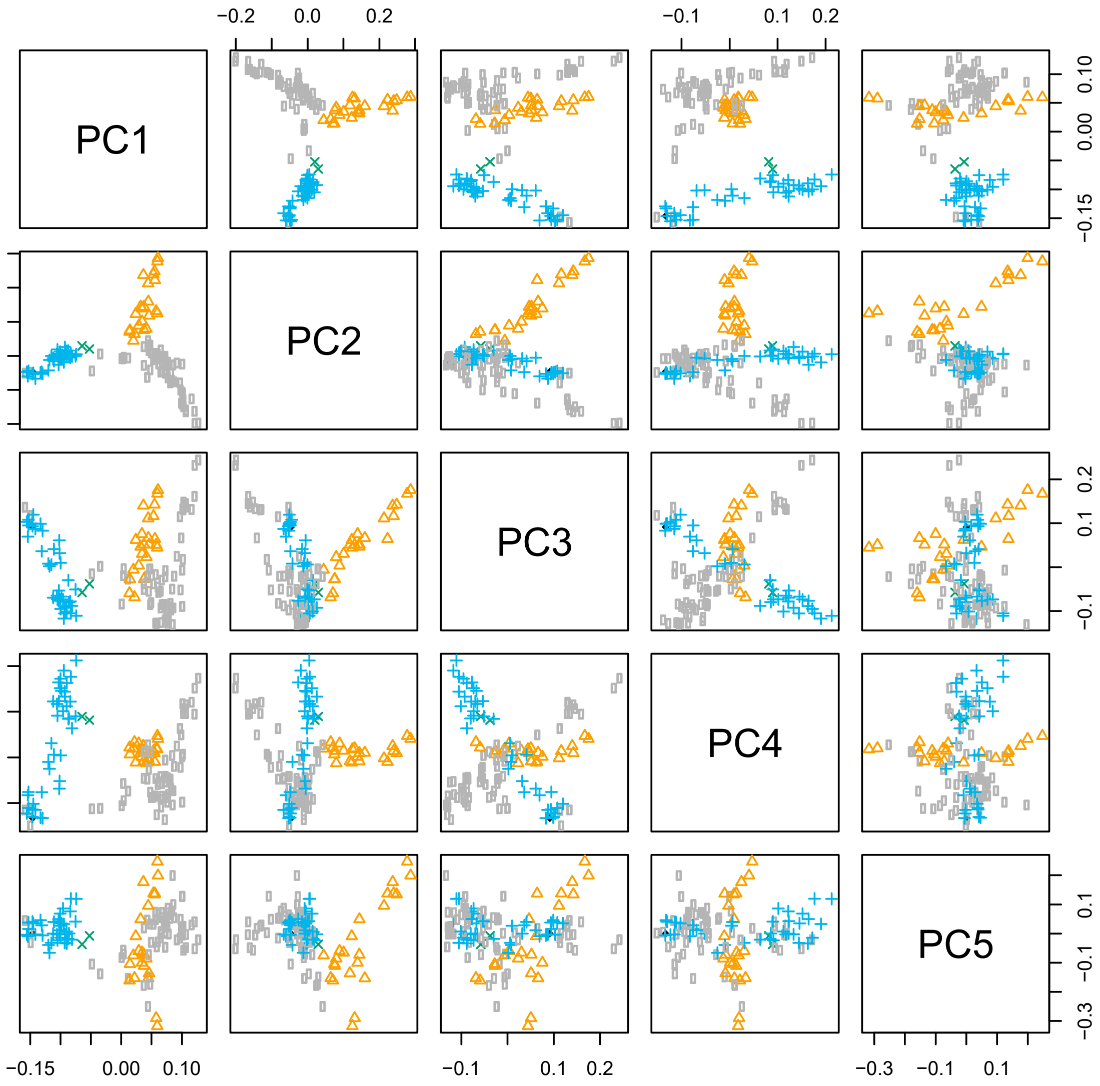

Figure 3. Evidence of population structure in results from a principal component analysis (PCA). Each point on the plot represents an individual horse. Points coloured according to breed: grey circles = Arabians (n = 61); orange triangles = Persian Horses (n = 23); blue '+' = Quarter Horses (n = 39); green 'x' = Standardbreds (n = 2); black diamond = Thoroughbreds (n = 1).

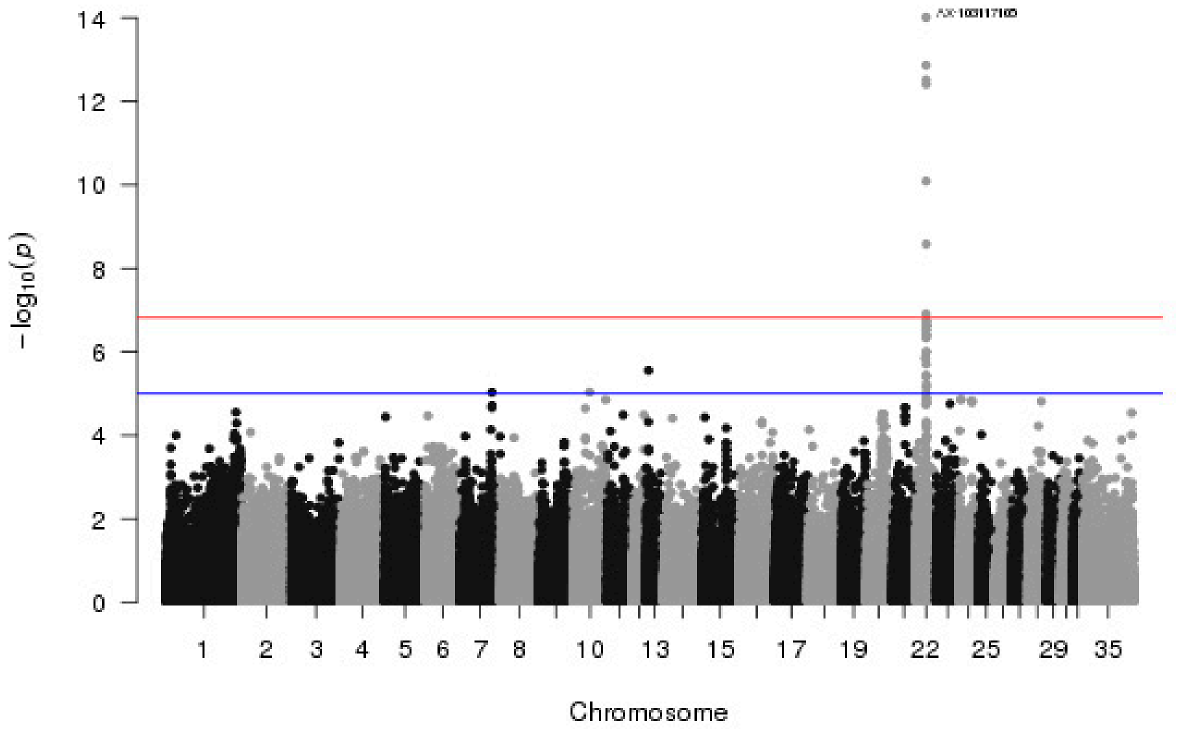

Figure S4. Manhattan plot showing primary GWAS result. Linear model run in Plink with 10 principal components and genotype at AX-103951024 and AX-104805525 fitted as covariates.

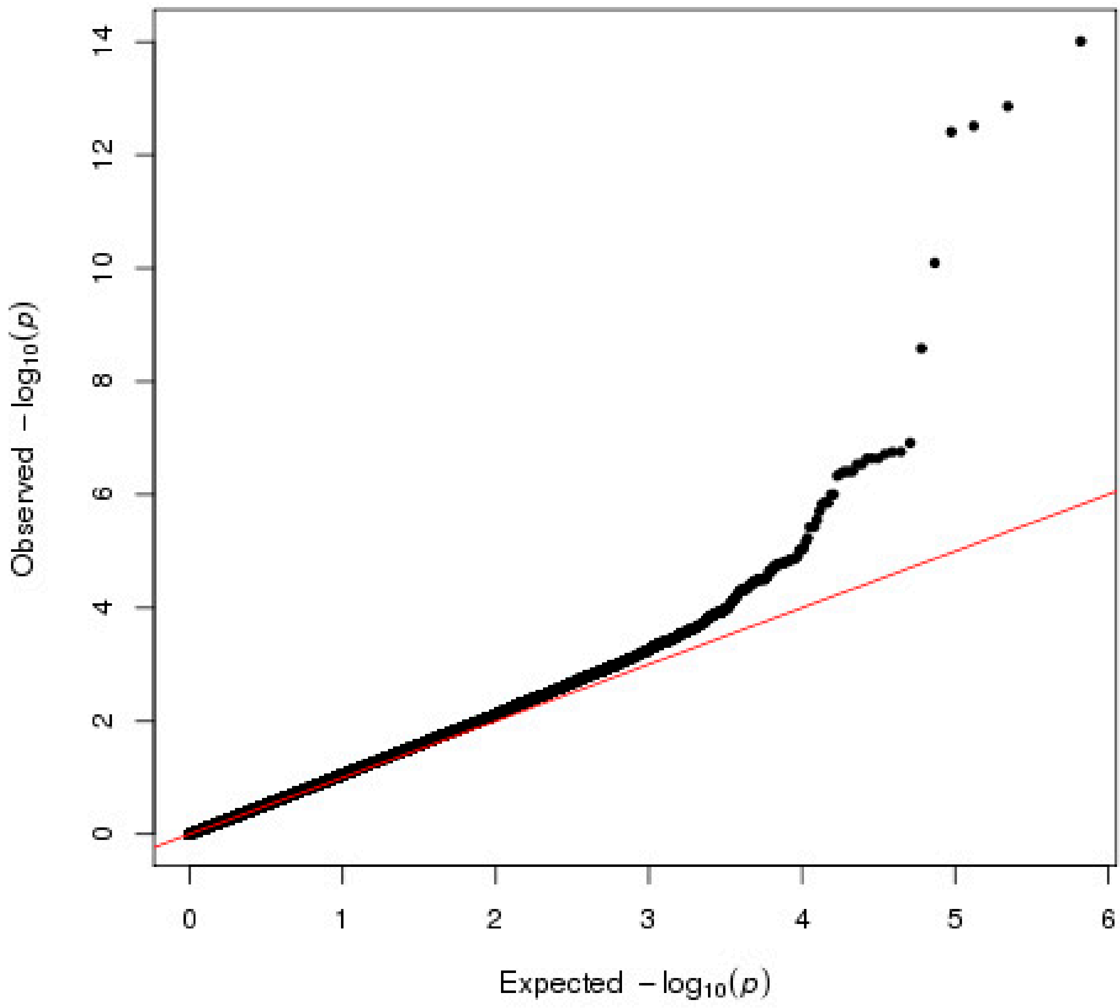

Figure S5. QQ plot showing primary GWAS result. Linear model run in Plink with 10 principal components and genotype at AX-103951024 and AX-104805525 fitted as covariates. Lambda = 1.07.

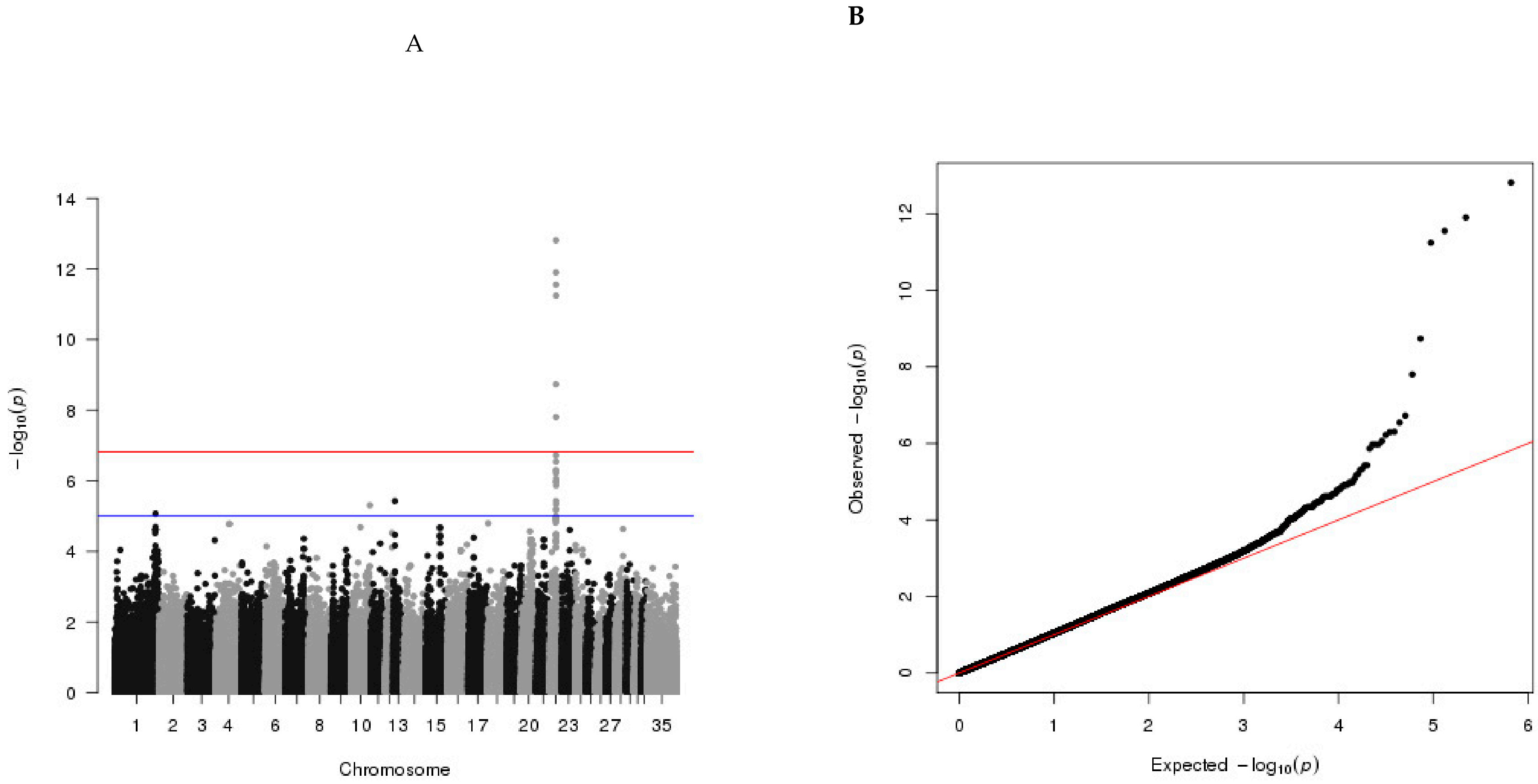

Figure S6 Results from a sensitivity analysis. GWAS conducted after having excluded one of each pair whose genetic relatedness was estimated to be >0.4 (N=114). (**A**) Manhattan plot; (**B**) QQ plot (lambda = 1.05).

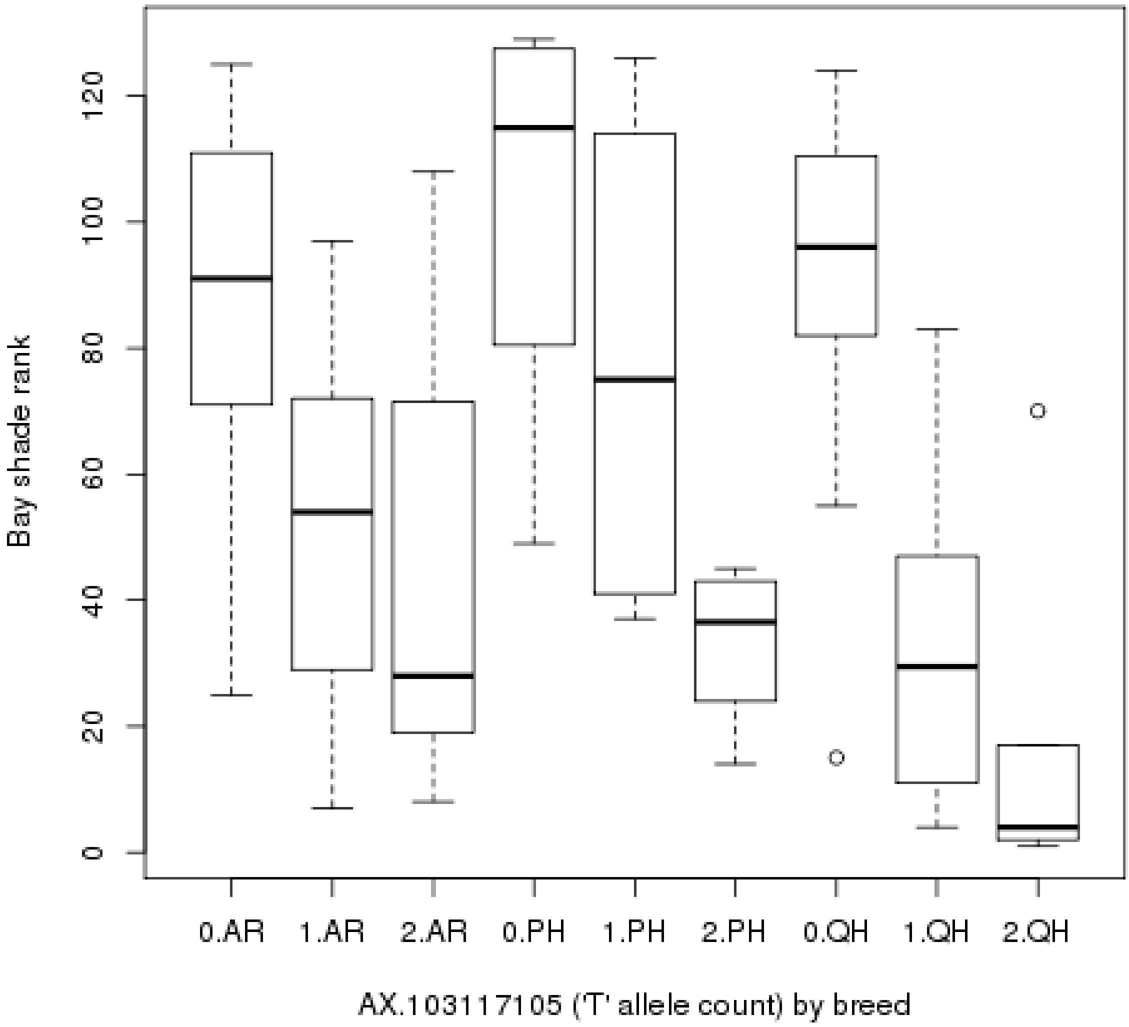

Figure S7 Effect of AX-103117105 on shade of bay by breed group. AR = Arabians (*n* = 61); PH = Persian Horses (*n* = 23); QH = Quarter Horses (*n* = 39). Upper and lower hinges of boxplots correspond to the first and third quartiles, with the centre line indicating the median and whiskers extending from the hinge to the largest (smallest) value no further than 1.5 x IQR from the hinge; outliers beyond this limit are plotted as unfilled points.

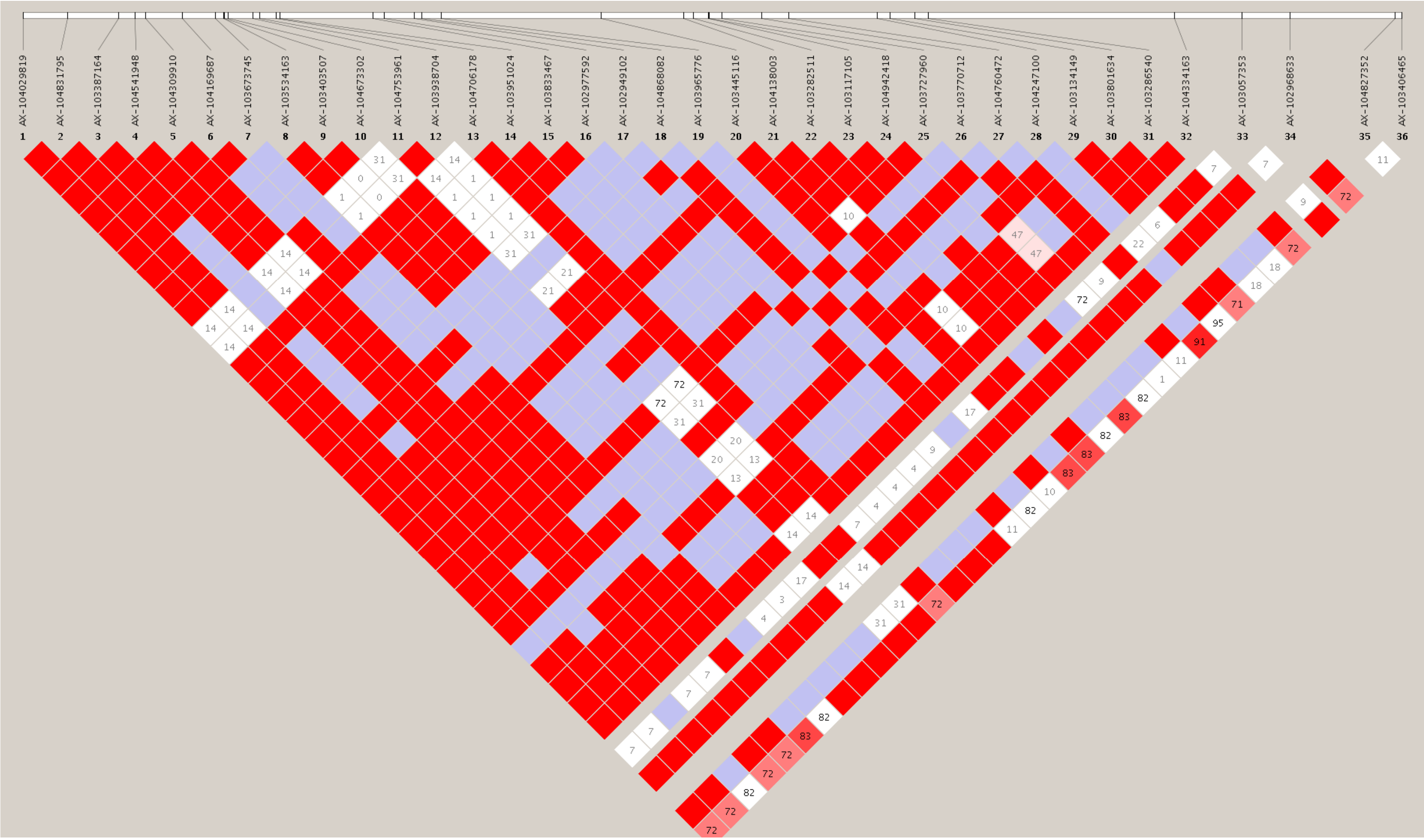

Figure S8. Haploview plot [7] of associated region using Arabian samples (*N* = 61). All SNPs within 200,000 bp up and downstream of the lead SNP (AX-103117105) included. D' values and confidence levels (logarithm of odds, LOD) are represented as red for D' = 1, LOD>2; shades of pink for high D', LOD>2; white for D'<1, LOD<2; blue for D' = 1, LOD<2.

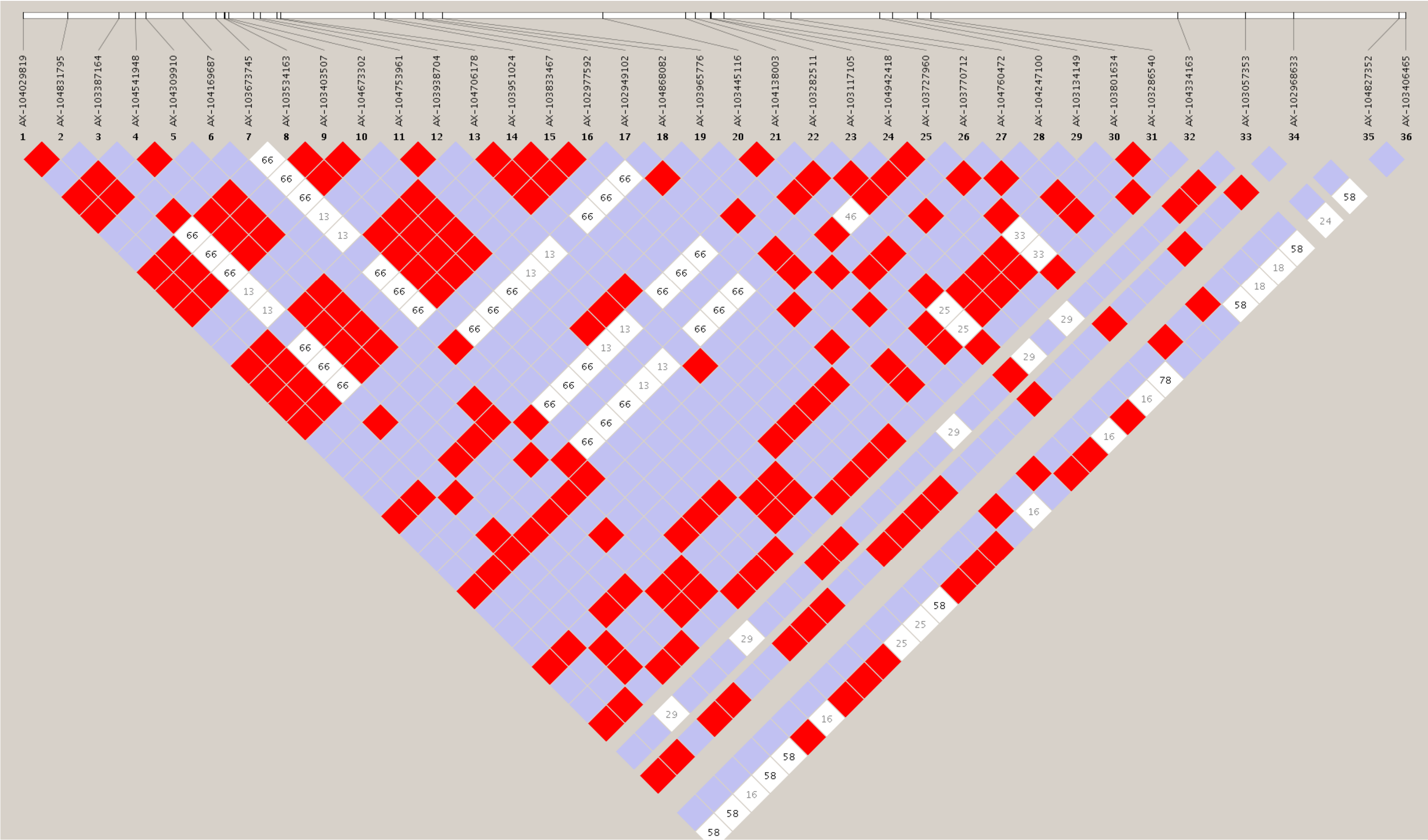

Figure S9. Haploview plot [7] of associated region using Persian Horse samples (*N* = 23). All SNPs within 200,000bp up and downstream of the lead SNP (AX-103117105) included. D' values and confidence levels (logarithm of odds, LOD) are represented as red for D' = 1, LOD>2; shades of pink for high D', LOD>2; white for D'<1, LOD<2; blue for D' = 1, LOD<2.

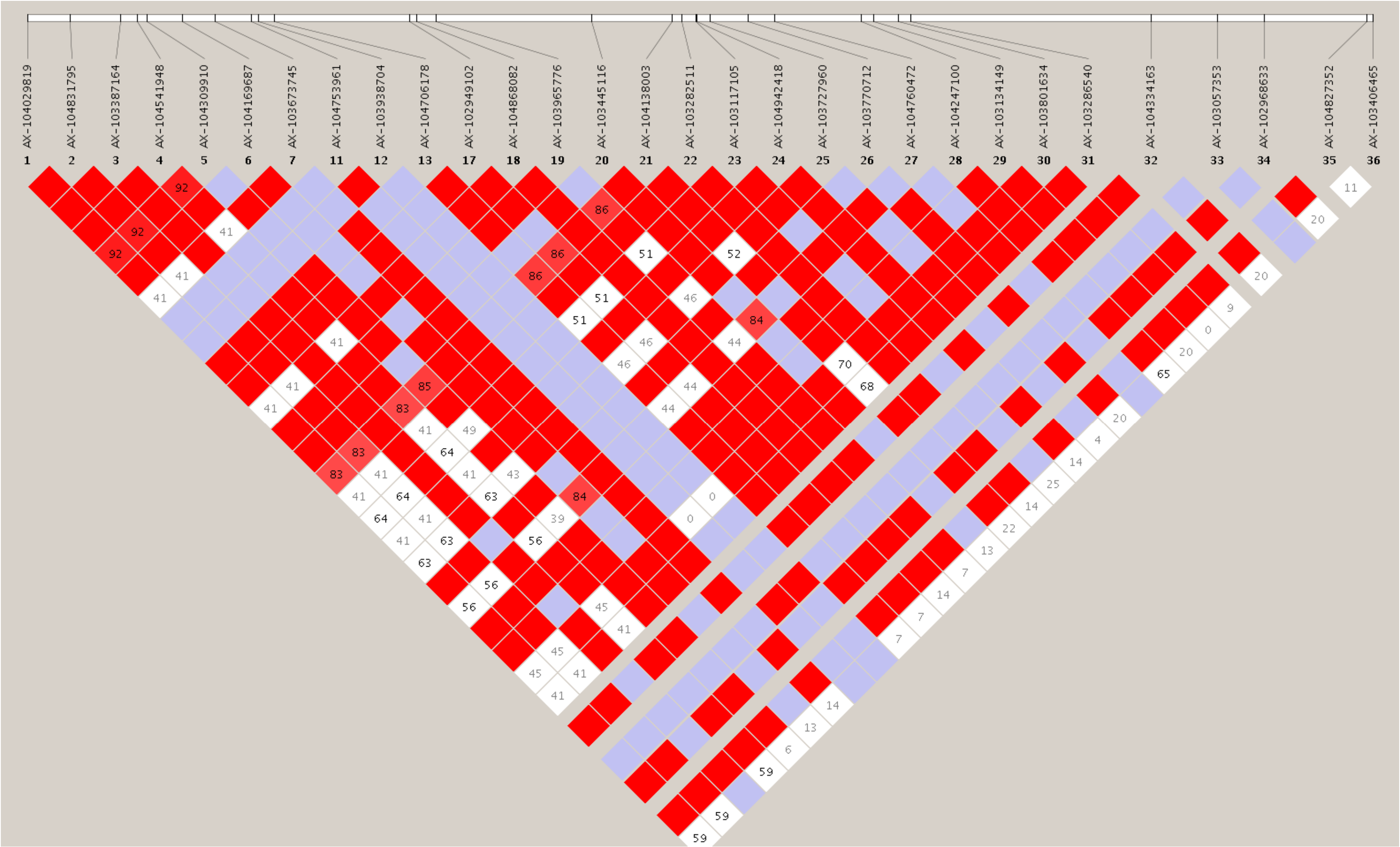

Figure S10. Haploview plot [7] of associated region using Quarter Horse samples (*N* = 39). All SNPs within 200,000bp up and downstream of the lead SNP (AX-103117105) included. D' values and confidence levels (logarithm of odds, LOD) are represented as red for D' = 1, LOD>2; shades of pink for high D', LOD>2; white for D'<1, LOD<2; blue for D' = 1, LOD<2.

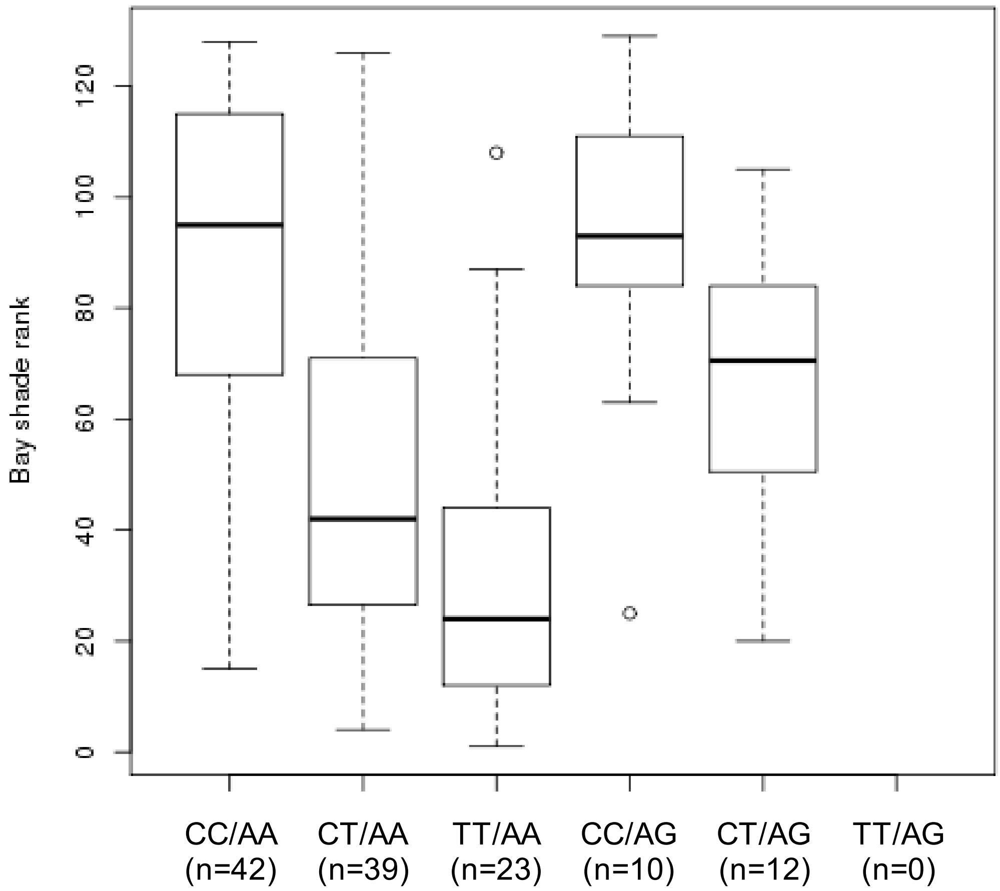

Figure S11. The combined effect of AX-103117105 (T/C) and AX-103951024 (A/G) genotypes on shade of bay. The first genotype is for AX-103117105 and the second is for AX-103951024. Upper and lower hinges of boxplots correspond to the first and third quartiles, with the centre line indicating the median and whiskers extending from the hinge to the largest (smallest) value no further than 1.5 x IQR from the hinge; outliers beyond this limit are plotted as unfilled points.

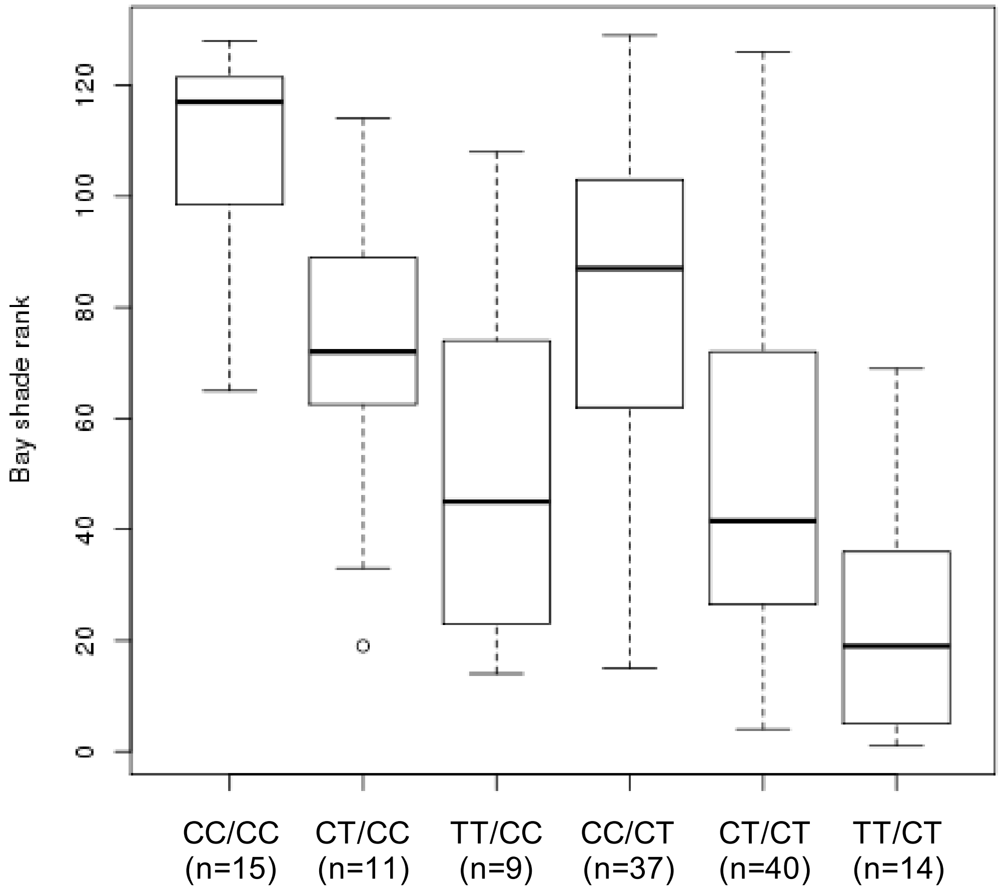

Figure 12. The combined effect of AX-103117105 (T/C) and AX-104805525 (T/C) genotypes on shade of bay. The first genotype is for AX-103117105 and the second is for AX-104805525. Upper and lower hinges of boxplots correspond to the first and third quartiles, with the centre line indicating the median and whiskers extending from the hinge to the largest (smallest) value no further than 1.5 x IQR from the hinge; outliers beyond this limit are plotted as unfilled points.

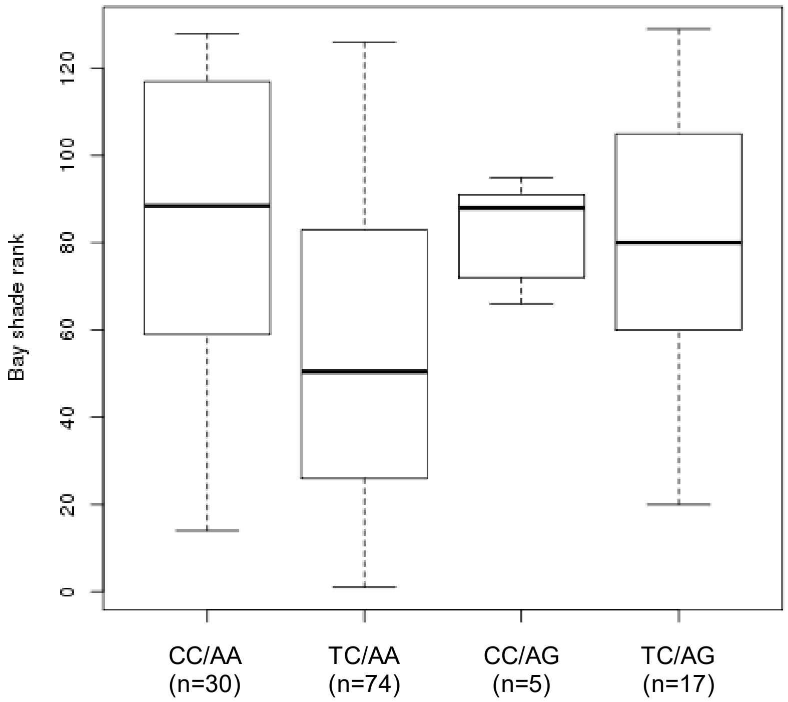

Figure S13 The combined effect of AX-104805525 (T/C) and AX-103951024 (A/G) genotypes on shade of bay. The first genotype is for AX-104805525 and the second is for AX-103951024. Upper and lower hinges of boxplots correspond to the first and third quartiles, with the centre line indicating the median and whiskers extending from the hinge to the largest (smallest) value no further than 1.5 x IQR from the hinge; outliers beyond this limit are plotted as unfilled points.

Supplementary References

1. Patterson Rosa, L.; Walker, N.L.; Mallicote, M.M.; MacKay, R.J.; Brooks, S.A. Genomics of Congenital Idiopathic Anhidrosis in the Stock-Type Horse. *Equine Veterinary Journal,* **2020,** in review.
2. Cosgrove, E.J.; Sadeghi, R.; Schlamp, F.; Holl, H.M.; Moradi-Shahrbabak, M.; Miraei-Ashtiani, S.R.; Abdalla, S.; Shykind, B.; Troedsson, M.; Stefaniuk-Szmukier, M., et al., Genome diversity and the origin of the Arabian horse. *Scientific Reports* **2020**, *In Press*.
3. Schaefer, R.J.; Schubert, M.; Bailey, E.; Bannasch, D.L.; Barrey, E.; Bar-Gal, G.K.; Brem, G.; Brooks, S.A.; Distl, O.; Fries, R.; et al. Developing a 670k genotyping array to tag ~2M SNPs across 24 horse breeds. *BMC Genomics* **2017**, *18*, 565.
4. Rieder, S.; Taourit, S.; Mariat, D.; Langlois, B.; Guérin, G. Mutations in the agouti (ASIP), the extension (MCIR), and the brown (TYRP1) loci and their association to coat color phenotypes in horses (Equus caballus). *Mammalian Genome*. **2001**;12(6):450-455. doi:10.1007/s003350020017
5. Druml, T.; Grilz-Seger, G.; Horna, M.; Brem, G. Discriminant Analysis of Colour Measurements Reveals Allele Dosage Effect of ASIP/MC1R in Bay Horses. *Czech J. Anim. Sci.* **2018**, *63*, 347–355.
6. Yang, J.; Lee, S.H.; Goddard, M.E.; Visscher, P.M. GCTA: A tool for genome-wide complex trait analysis. *Am. J. Hum. Genet.* **2011**, *88*, 76–82.
7. Barrett, J.C.; Fry, B.; Maller, J.; Daly, M.J. Haploview: Analysis and visualization of LD and haplotype maps. *Bioinformatics* **2005**, *21*, 263–265.

© 2020 by the authors. Submitted for possible open access publication under the terms and conditions of the Creative Commons Attribution (CC BY) license (http://creativecommons.org/licenses/by/4.0/).
